# The Association between Respiratory Infection and Air Pollution in the Setting of Air Quality Policy and Economic Change

**DOI:** 10.1513/AnnalsATS.201810-691OC

**Published:** 2019-03

**Authors:** Daniel P. Croft, Wangjian Zhang, Shao Lin, Sally W. Thurston, Philip K. Hopke, Mauro Masiol, Stefania Squizzato, Edwin van Wijngaarden, Mark J. Utell, David Q. Rich

**Affiliations:** ^1^ Division of Pulmonary and Critical Care Medicine; ^3^ Department of Biostatistics and Computational Biology; ^4^ Department of Public Health Sciences, and; ^6^ Department of Environmental Medicine, University of Rochester Medical Center, Rochester, New York; ^2^ Department of Environmental Health Sciences, University at Albany, State University of New York, Albany, New York; and; ^5^ Institute for a Sustainable Environment, and Center for Air Resources Engineering and Science, Clarkson University, Potsdam, New York

**Keywords:** particulate matter, bacterial pneumonia, influenza virus, air pollution

## Abstract

**Rationale:** Fine particulate matter air pollution of 2.5 μm or less in diameter (PM_2.5_) has been associated with an increased risk of respiratory disease, but assessments of specific respiratory infections in adults are lacking.

**Objectives:** To estimate the rate of respiratory infection healthcare encounters in adults associated with acute increases in PM_2.5_ concentrations.

**Methods:** Using case–crossover methods, we studied 498,118 adult New York State residents with a primary diagnosis of influenza, bacterial pneumonia, or culture-negative pneumonia upon hospitalization or emergency department (ED) visit (2005–2016). We estimated the relative rate of healthcare encounters associated with increases in PM_2.5_ in the previous 1–7 days and explored differences before (2005–2007), during (2008–2013), and after (2014–2016) implementation of air quality policies and economic changes.

**Results:** Interquartile range increases in PM_2.5_ over the previous 7 days were associated with increased excess rates (ERs) of culture-negative pneumonia hospitalizations (2.5%; 95% confidence interval [CI], 1.7–3.2%) and ED visits (2.5%; 95% CI, 1.4–3.6%), and increased ERs of influenza ED visits (3.9%; 95% CI, 2.1–5.6%). Bacterial pneumonia hospitalizations, but not ED visits, were associated with increases in PM_2.5_ and, though imprecise, were of a similar magnitude to culture-negative pneumonia (Lag Day 6 ER, 2.3%; 95% CI, 0.3–4.3). Increased relative rates of influenza ED visits and culture-negative pneumonia hospitalizations were generally larger in the “after” period (*P* < 0.025 for both outcomes), compared with the “during” period, despite reductions in overall PM_2.5_ concentrations.

**Conclusions:** Increased rates of culture-negative pneumonia and influenza were associated with increased PM_2.5_ concentrations during the previous week, which persisted despite reductions in PM_2.5_ from air quality policies and economic changes. Though unexplained, this temporal variation may reflect altered toxicity of different PM_2.5_ mixtures or increased pathogen virulence.

Influenza and bacterial pneumonia are a leading cause of adult morbidity and mortality in the United States (1), and their risk factors are actively being studied (2, 3). Although fine particulate matter air pollution of 2.5 μm or less in diameter (PM_2.5_) has previously been associated with general cardiopulmonary morbidity and mortality worldwide (4), studies have also identified it as a potential contributor to respiratory infection in both adults and children (5–9), specifically, influenza (5, 6) and bacterial pneumonia (7, 9). An increased risk of respiratory infection associated with PM_2.5_ exposure has been reported in Utah (6, 8), and in a study of four other U.S. cities (10). Recent studies examined PM_2.5_ associations with respiratory viral infections in children (0–17 yr old) and adults (≥18 yr old) (6), lower respiratory infection severity in older adults (≥65) (8), and respiratory disease in general, including pneumonia in children and adults (10). Although specific respiratory infections (e.g., respiratory syncytial virus) have been studied in children, it is unclear whether specific respiratory infections (e.g., culture-negative pneumonia, influenza, and bacterial pneumonia) in adults are associated with short-term PM_2.5_ exposure.

Changes in the ambient concentration and composition of PM_2.5_ provide a unique opportunity to investigate whether these changes in ambient PM_2.5_ composition result in changes in the rate of respiratory infection associated with each IQR increase in PM_2.5_ concentration (i.e., is the same PM_2.5_ mass more or less toxic?). Policy initiatives to improve air quality in New York State and the Northeast Region of the United States over the past 10 years were summarized by Squizzato and colleagues (11). These initiatives included requirements for ultralow sulfur (<15 ppm S) fuel for on-road and nonroad diesel, and after July 1, 2012, for home heating. Pollution controls were required for on-road, heavy-duty, diesel vehicles. Ontario, Canada has closed its coal-fired power plants, and emission controls were installed on Ohio River Valley plants, reducing NOx and SO_2_ emissions upwind of New York State. The 2008 recession and the change in relative prices of natural gas and coal and oil resulted in a shift in generation to gas (11). PM_2.5,_ NO_x_, and SO_2_ concentrations have decreased across New York State after these policies were implemented and the 2008 recession occurred (11). However, the composition of PM changed, with increased concentrations of secondary organic carbon (SOC) (12), which might modify the rate of respiratory infections associated with PM_2.5_.

To address the relative lack of studies of specific respiratory infections in adults, we used a large, multiyear (2005–2016) New York state–wide database to separately estimate the rates of hospital admissions and emergency department (ED) visits for influenza, bacterial pneumonia, and culture-negative pneumonia, among New York adult residents, associated with short-term increases in mean PM_2.5_ concentrations in the previous 1–7 days. We hypothesized that increased PM_2.5_ concentrations would be associated with increased rates of all outcomes. We then explored whether each relative rate differed before (2005–2007), during (2008–2013), and after (2014–2016) the changes in air quality described previously here.

## Methods

### Study Population

From the SPARCS (Statewide Planning and Research Cooperative System) database, respiratory infection hospital admissions and ED visits (patients treated and released home) were retained for all adult New York residents (≥18 yr of age) who lived within 15 miles of the Buffalo, Rochester, Albany, Bronx, Manhattan, or Queens, New York, PM_2.5_ monitoring sites from January 1, 2005 to December 31, 2016 (*n* = 319,570 hospitalizations and *n* = 178,548 ED visits were available for analysis). We included subjects with a primary diagnosis (at time of hospitalization or ED visit) of influenza (*International Classification of Disease* [ICD] *9* = 487.0, 487.8, 488.0, 488.01, 488.02, 488.1, 488.11, 488.12, 488.8, 488.81, 488.82; ICD10 = J09, J09.X1, J09.X2, J10.0, J10.00, J10.01, J10.08, J10.1, J11.0, J11.00, J11.08, J11.1), bacterial pneumonia (ICD9 = 481, 482, 483.0, 483.1; ICD10 = J13, J14, J15, J16, A48.1), or culture-negative pneumonia (ICD9 = 485, 486; ICD10 = J18). Culture-negative pneumonia is a common diagnosis, as modern culture techniques only identify a causative pathogen in under 50% of the patients diagnosed with pneumonia (13, 14). Culture-negative pneumonia is best viewed as an undifferentiated infection, as it can be bacterial or viral in origin. This study was reviewed and approved by the Institutional Review Board at the University at Albany, State University of New York.

### Air Pollution and Weather

Hourly PM_2.5_ concentrations at the six urban air-monitoring stations (Buffalo, Rochester, Albany, the Bronx, Manhattan, and Queens, NY) were retrieved from the U.S. Environmental Protection Agency (https://aqs.epa.gov/api). Further details on measurement of PM_2.5_, temperature, and relative humidity have been described previously (12). For each subject, daily PM_2.5_, temperature, and relative humidity values were assigned from the monitoring station closest to their residence.

### Statistical Analysis

To estimate the rate of respiratory infection hospital admissions and ED visits associated with each interquartile range (IQR) increase in PM_2.5_ concentration on the same day (Lag Day 0), we used a time-stratified, case–crossover design (15, 16). For all influenza hospital admissions from all six urban sites (assuming a common slope across sites), we fit a conditional logistic regression model stratified on each respiratory infection hospital admission matched set (one case and three to four control periods per subject), and regressed case–control status (i.e., case = 1, control = 0) against the mean PM_2.5_ concentration on case and control days. Because case periods and their matched control periods are derived from the same person, and a conditional analysis is conducted, non–time-varying confounders, such as underlying medical conditions, long-term time trends, and season, are controlled by design. As is standard in case–crossover studies, from this statistical model, the odds ratio is a direct estimate of the rate ratio and its 95% confidence interval (CI). The excess rate (ER) is the percent increase in the rate per unit of exposure (i.e., [rate ratio − 1.0] × 100%).

We included natural splines for temperature and relative humidity (4 *df*), which were determined using Akaike’s information criterion (17). This same model was run for the PM_2.5_ means of Lag Days 0–1, 0–2, 0–3, 0–4, 0–5, and 0–6, and then separately for ED visits and hospitalizations for influenza, bacterial pneumonia, and culture-negative pneumonia. Because we examined seven lag times for each disease subgroup, statistical significance for slopes was defined as *P* less than 0.007 (0.05/7). The lag times averaged over increasing lag days rather than segmental lag days (i.e., Day 1, Day 2, Day 3) were used to more accurately view the ER pattern across lag times. In the Results section, we present the largest lagged effect, and then describe whether other lag times had similar effects. Furthermore, inference was made considering several factors, including the pattern of response across these lag averaging times, the precision of each estimate, as well as statistical significance.

Next, we explored whether the association between PM_2.5_ and each respiratory infection admission rate differed by period (before = 2005–2007, during = 2008–2013, after = 2014–2016), by adding indicator variables for period and two interaction terms of period and PM_2.5_ to the model. The significance of the difference of the PM_2.5_ effect across periods was evaluated by a 2-*df* test for interaction. If statistically significant, we examined whether the ER in the after period was different from the ER in the during and before periods, using a *P* value of 0.025 to define statistical significance. All analyses were done using R version 3.0.1 (https://www.r-project.org/).

## Results

Subjects who were hospitalized or required ED evaluation for respiratory infection were predominantly older in the before period (mean age, 65 yr) compared with the during and after periods (59 yr old). The race/ethnicity of the subject population changed from the before to the after period with a decreased proportion of white subjects (54% down to 43%) and a larger proportion of black and Hispanic subjects (24% up to 27% and 15% up to 18%, respectively). The race and sex of subjects (53–54% female) remained stable across the multiple periods.

**Figure 1.**
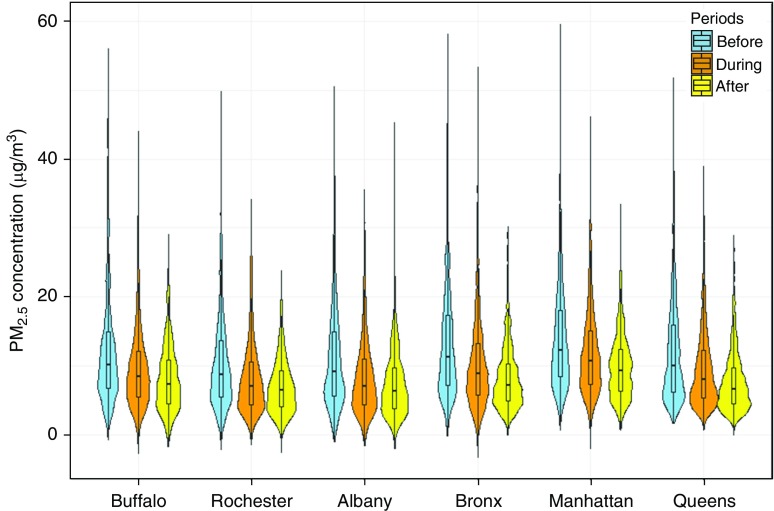
Distribution of fine particulate matter air pollution of 2.5 μm or less in diameter (PM_2.5_) concentrations (μg/m^3^) for case periods by study site and time period.

The majority of patients in the before, during, and after periods had health care encounters (hospitalizations or ED visits) for culture-negative pneumonia (89%, 77%, and 68%, respectively). The most common comorbidities for subjects included hypertension (20–38%), fluid and electrolyte disorders (15–28%), diabetes (13–22%), and heart failure (13–22%) (Table 1). The mean (±SD) length of stay for subjects decreased from the before period (5.6 d) to the after period (3.3 d) (Table 1). The overall number of yearly hospitalizations steadily decreased (from 34,458 to 21,735), whereas the number of ED visits increased (from 9,799 to 20,874) from 2005 to 2016. The distributions of PM_2.5_ concentrations for case and control periods are shown in Figure 1 and in Table E1 in the online supplement. Overall, case and control period concentrations were similar. We did not observe a difference in the seasonal patterns for respiratory infection hospitalizations or ED visits from 2005 to 2016 (*see* Table E2). For influenza, the highest proportion of admissions and ED visits were present in winter and spring, whereas the lowest proportion of admissions was present in summer and fall.

**Table 1.** Characteristics of respiratory infectious hospital admissions and emergency department visits (2005–2016), by study site/city

Characteristic	Before (*n* = *125,817*)		During (*n* = *250,845*)		After (*n* = *121,456*)	
	*n*	%	*n*	%	*n*	%
Infection						
Culture-negative pneumonia	111,450	89	193,713	77	83,060	68
Influenza	6,057	5	38,745	15	27,484	23
Bacterial pneumonia	8,310	7	18,387	7	10,912	9
Male	59,377	47	116,344	46	55,530	46
Age, yr, mean (SD)	65 (21)		59 (22)		59 (21)	
≥18–39	18,016	14	54,975	22	26,979	22
≥40–49	14,296	11	30,407	12	14,092	12
≥50–59	15,689	12	36,159	14	19,416	16
≥60–69	16,988	14	34,098	14	18,126	15
≥70–79	23,160	18	36,110	14	17,173	14
≥80	37,668	30	59,096	24	25,670	21
Race/ethnicity						
White	67,804	54	121,693	49	52,563	43
Black	30,181	24	65,887	26	32,924	27
American Indian	790	1	1,267	1	335	—
Asian	4,150	3	9,945	4	—	—
Native Hawaiian	47	—	161	—	—	—
Hispanic	18,260	15	43,293	17	21,699	18
Year						
2005	44,257	35	—	—	—	—
2006	41,569	33	—	—	—	—
2007	39,991	32	—	—	—	—
2008	—	—	40,894	16	—	—
2009	—	—	51,487	21	—	—
2010	—	—	36,711	15	—	—
2011	—	—	39,736	16	—	—
2012	—	—	39,260	16	—	—
2013	—	—	42,757	17	—	—
2014	—	—	—	—	40,412	33
2015	—	—	—	—	38,438	32
2016	—	—	—	—	42,606	35
Season						
Spring	27,126	22	57,659	23	22,744	19
Summer	33,880	27	61,976	25	35,484	29
Fall	25,263	20	49,191	20	20,553	17
Winter	39,548	31	82,019	33	42,675	35
Length of stay, d, mean (SD)	5.6 (9.0)		4.3 (8.2)		3.3 (5.6)	
Comorbidity						
Hypertension	47,285	38	83,298	33	24,610	20
Fluid, electrolyte, acid–base	35,774	28	64,010	26	17,990	15
Diabetes mellitus	28,161	22	53,119	21	16,164	13
Heart failure	25,652	20	54,113	22	15,616	13
Disorders of lipoid metabolism	17,523	14	46,396	19	16,689	14
Other chronic heart disease	22,705	18	39,104	16	10,642	9
Cardiac arrhythmia	21,918	17	38,262	15	11,269	9
Cardiac pacemaker	15,035	12	34,835	14	12,665	10
Anemia	15,964	13	31,175	12	8,598	7
Asthma	14,260	11	31,187	12	9,856	8
Other diseases of the lung	12,535	10	28,939	12	8,204	7
Nondependent abuse of drugs	9,996	8	27,455	11	9,923	8
Dyspnea	7,265	6	27,688	11	9,232	8
Exposures to health hazards	6,158	5	24,215	10	13,482	11
Constitutional Symptoms	9,898	8	23,967	10	9,634	8

*Definition of abbreviation*: SD = standard deviation.

**Figure 2.**
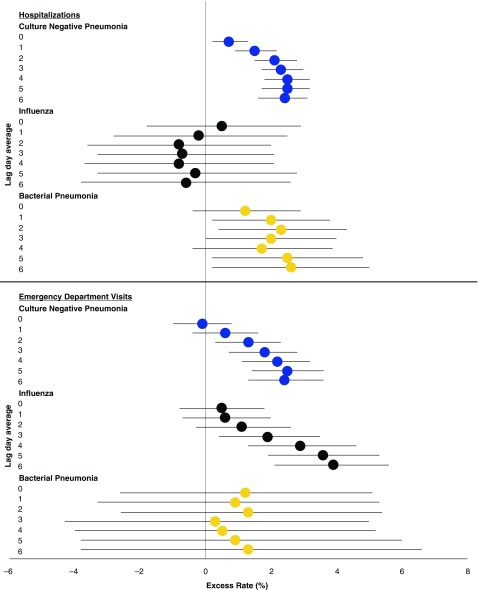
Excess rate (and 95% confidence interval [CI]) of infectious disease hospitalizations and emergency department visits associated with each interquartile range increase in concentration of fine particulate matter air pollution of 2.5 μm or less in diameter, by infection and lag day mean.

For culture-negative pneumonia, IQR increases in PM_2.5_ concentration in the previous 2–7 days (Lag Days 0–1, 0–2, 0–3, 0–4, 0–5, and 0–6) were associated with increased rates of hospitalizations, with the largest in the previous 5 days (ER, 2.5%; 95% CI, 1.8–3.2%) and 6 days (ER, 2.5%; 95% CI, 1.7–3.2%) (Figure 2; Table 2). Similarly, IQR increases in PM_2.5_ in the previous 4–7 days were associated with increased rates of culture-negative pneumonia ED visits, with the largest in the previous 6 days (ER, 2.5%; 95% CI, 1.4–3.6%). Although there were no associations between PM_2.5_ and influenza hospitalizations, IQR increases in the previous 5–7 days were associated with increased rates of ED visits for influenza, with the largest at 7 days (ER, 3.9%; 95% CI, 2.1–5.6%). Though imprecise due to low sample size, the ER of bacterial pneumonia hospitalizations in the previous 1–7 days were of similar magnitude to those of culture-negative pneumonia (Lag Day 6 ER, 2.3%; 95% CI, 0.3–4.3). No association was observed between PM_2.5_ and ED visits for bacterial pneumonia (Table 2 and Figure 2).

**Table 2.** Excess rate of respiratory infectious hospital admissions and emergency department visits associated with interquartile range increases in fine particulate matter air pollution of 2.5 μm or less in diameter concentration, by lag time, and outcome*

Outcome	Lag Days	Hospital Admissions				Emergency Department Visits			
		IQR (μg/m^3^)	*n* Cases	Excess Rate % (95% CI)	*P* Value	IQR (μg/m^3^)	*n* Cases	Excess Rate % (95% CI)	*P* Value
Culture-negative pneumonia	0	7	267,905	0.7 (0.2 to 1.3)	0.01	6.6	110,982	−0.1 (−1.0 to 0.8)	0.78
	0–1	6.5	269,968	1.5 (0.9 to 2.2)	<0.001	6.1	112,113	0.6 (−0.4 to 1.6)	0.22
	0–2	6.2	272,191	2.1 (1.5 to 2.8)	<0.001	5.8	113,187	1.3 (0.3 to 2.3)	0.01
	0–3	5.7	272,681	2.3 (1.7 to 3.0)	<0.001	5.3	113,382	1.8 (0.7 to 2.8)	<0.001
	0–4	5.5	273,017	2.5 (1.8 to 3.2)	<0.001	5.1	113,521	2.2 (1.1 to 3.2)	<0.001
	0–5	5.2	273,266	2.5 (1.7 to 3.2)	<0.001	4.9	113,623	2.5 (1.4 to 3.6)	<0.001
	0–6	5	273,430	2.4 (1.6 to 3.1)	<0.001	4.8	113,679	2.4 (1.3 to 3.6)	<0.001
Influenza	0	6.4	14,072	0.5 (−1.8 to 2.9)	0.67	6.7	55,819	0.5 (−0.8 to 1.8)	0.47
	0–1	6.5	14,388	−0.2 (−2.8 to 2.5)	0.88	6.4	56,573	0.6 (−0.7 to 2.0)	0.36
	0–2	6.4	14,690	−0.8 (−3.6 to 2.0)	0.56	6.2	57,308	1.1 (−0.3 to 2.6)	0.14
	0–3	5.7	14,729	−0.7 (−3.3 to 2.1)	0.63	6	57,365	1.9 (0.4 to 3.5)	0.01
	0–4	5.6	14,738	−0.8 (−3.7 to 2.1)	0.58	5.9	57,399	2.9 (1.3 to 4.6)	<0.001
	0–5	5.5	14,750	−0.3 (−3.3 to 2.8)	0.86	5.7	57,430	3.6 (1.9 to 5.3)	<0.001
	0–6	5.5	14,758	−0.6 (−3.8 to 2.6)	0.71	5.4	57,455	3.9 (2.1 to 5.6)	<0.001
Bacterial pneumonia	0	6.5	29,774	1.1 (−0.4 to 2.7)	0.16	6.2	6,862	1.6 (−1.9 to 5.1)	0.37
	0–1	6	30,066	1.9 (0.2 to 3.6)	0.03	6	6,925	1.4 (−2.4 to 5.3)	0.48
	0–2	5.7	30,378	2.1 (0.4 to 3.9)	0.02	5.2	7,002	1.5 (−2.2 to 5.3)	0.44
	0–3	5.3	30,442	1.8 (0.0 to 3.6)	0.05	5.5	7,007	0.2 (−4.0 to 4.5)	0.94
	0–4	5.2	30,484	1.7 (−0.2 to 3.6)	0.07	5	7,010	0.0 (−4.1 to 4.3)	0.99
	0–5	5.2	30,509	2.3 (0.3 to 4.3)	0.03	5	7,019	0.4 (−4.0 to 5.0)	0.87
	0–6	5	30,518	2.1 (0.1 to 4.2)	0.04	5	7,020	0.5 (−4.2 to 5.4)	0.83

*Definition of abbreviations*: CI = confidence interval; IQR = interquartile range.

* Models adjusted for temperature (4 *df*) and relative humidity using natural splines (4 *df*).

**Figure 3.**
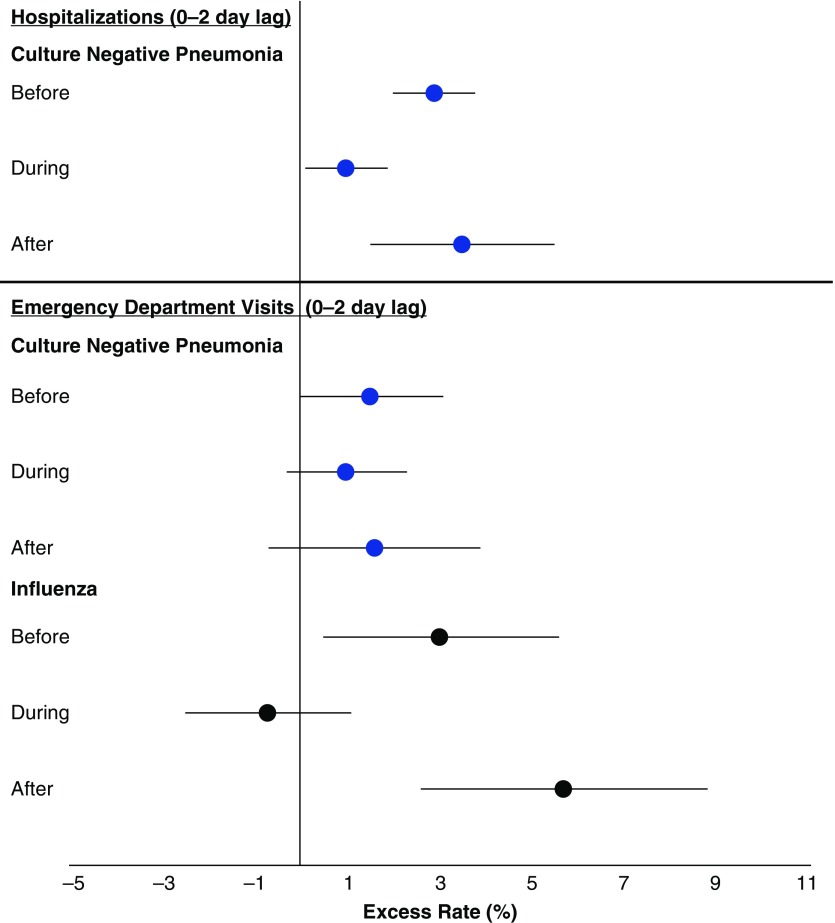
Excess rate (and 95% confidence interval [CI]) of hospitalizations and emergency department visits for culture-negative pneumonia and influenza associated with each interquartile range increase in concentration of fine particulate matter air pollution of 2.5 μm or less in diameter for the 0- to 2-day lag time by period.

Next, we explored whether the relative rates of hospitalizations for each outcome associated with IQR increases in ambient PM_2.5_ concentrations in the before, during, and after periods were different to determine whether changes in PM concentration and composition may have differentially triggered these respiratory infections (Table 3). The rate of culture-negative pneumonia hospitalizations, but not ED visits, associated with each 6.2-μg/m^3^ increase in PM_2.5_ concentration in the previous 3 days (Lag Days 0–2) was different across periods (*P* = 0.002), with the largest ERs in the before period (2.9%; 95% CI, 2.0–3.8%) and after period (3.5%; 95% CI, 1.5–5.5%) compared with the during period (1.0%; 95% CI, 0.1–1.9%) (Figure 3). There were also generally similar patterns in the previous 2, 4, and 5 days. Similarly, the increased rate of influenza ED visits associated with each 6.4-μg/m^3^ increase in PM_2.5_ concentration in the previous 3 days was different across periods (*P* < 0.001), with the largest ERs in the before period (3.0%; 95% CI, 0.5–5.6%) and after period (5.7%; 95% CI, 2.6–8.8%) compared with the during period (−0.7%; 95% CI, −2.5% to 1.1%) (Figure 3). There were similar patterns in the previous 1, 2, 4, and 5 days (Table 3).

**Table 3.** Excess rate of respiratory infectious hospital admissions associated with each interquartile range increase in concentration of fine particulate matter air pollution of 2.5 μm or less in diameter, by lag time, outcome, and time period

Outcome	Lag Day	Before (*n*)	During (*n*)	After (*n*)	IQR (*μg*/*m*^*3*^)	Before		During		After		*P* Value for Interaction (2 *df*)
						Excess Risk % (*95% CI*)	*P* Value	Excess Risk % (*95% CI*)	*P* Value	Excess Risk % (*95% CI*)	*P* Value	
Hospital admissions												
Culture-negative pneumonia	0	87,108	131,018	49,779	7.0	1.2 (0.4 to 1.9)	0.002	0.2 (−0.6 to 1.0)	0.67	1.0 (−0.7 to 2.7)	0.26	0.16
	0–1	87,347	132,663	49,958	6.5	2.2 (1.4 to 3.0)	<0.001	0.7 (−0.2 to 1.5)	0.14	2.1 (0.2 to 3.9)	0.03	0.02
	0–2	87,569	134,430	50,192	6.2	2.9 (2.0 to 3.8)	<0.001	1.0 (0.1 to 1.9)	0.04	3.5 (1.5 to 5.5)*	<0.001	0.002
	0–3	87,614	134,839	50,228	5.7	3.1 (2.1 to 4.0)	<0.001	1.3 (0.4 to 2.3)	0.01	3.7 (1.7 to 5.7)^†^	<0.001	0.01
	0–4	87,647	135,110	50,260	5.5	3.2 (2.2 to 4.2)	<0.001	1.5 (0.5 to 2.5)	0.00	3.7 (1.6 to 5.8)	0.001	0.02
	0–5	87,647	135,332	50,287	5.2	3.1 (2.1 to 4.1)	<0.001	1.6 (0.6 to 2.7)	0.00	3.4 (1.3 to 5.6)	0.002	0.06
	0–6	87,647	135,477	50,306	5.0	3.1 (2.1 to 4.2)	<0.001	1.5 (0.4 to 2.5)	0.01	2.8 (0.6 to 5.0)	0.01	0.06
Influenza	0	1,031	5,883	7,158	6.4	7.2 (1.5 to 13.1)	0.01	−0.8 (−3.5 to 1.9)	0.56	0.1 (−3.2 to 3.5)	0.95	0.03
	0–1	1,048	6,083	7,257	6.5	5.6 (−0.3 to 11.9)	0.07	−1.5 (−4.4 to 1.5)	0.33	−0.3 (−4.0 to 3.5)	0.86	0.08
	0–2	1,066	6,265	7,359	6.4	4.5 (−1.7 to 11.1)	0.16	−2.3 (−5.3 to 0.8)	0.14	−0.1 (−4.0 to 4.1)	0.98	0.07
	0–3	1,066	6,292	7,371	5.7	4.9 (−0.9 to 11.1)	0.10	−2.2 (−5.1 to 0.7)	0.14	0.3 (−3.5 to 4.3)	0.86	0.03
	0–4	1,066	6,295	7,377	5.6	4.8 (−1.2 to 11.1)	0.12	−2.4 (−5.4 to 0.7)	0.13	0.3 (−3.7 to 4.4)	0.89	0.03
	0–5	1,066	6,301	7,383	5.5	4.7 (−1.3 to 11.2)	0.13	−1.9 (−5.0 to 1.4)	0.26	1.3 (−2.9 to 5.6)	0.56	0.03
	0–6	1,066	6,306	7,386	5.5	4.2 (−2.0 to 10.8)	0.19	−2.2 (−5.5 to 1.3)	0.22	0.9 (−3.3 to 5.4)	0.67	0.04
Bacterial pneumonia	0	7,452	14,424	7,898	6.5	1.8 (−0.1 to 3.9)	0.07	−0.0 (−2.1 to 2.2)	0.99	2.1 (−1.6 to 6.0)	0.27	0.33
	0–1	7,503	14,629	7,934	6	2.3 (0.3 to 4.4)	0.03	1.4 (−0.9 to 3.7)	0.23	1.7 (−2.2 to 5.8)	0.39	0.78
	0–2	7,564	14,838	7,976	5.7	2.3 (0.3 to 4.5)	0.03	1.9 (−0.4 to 4.4)	0.11	1.7 (−2.3 to 5.9)	0.42	0.92
	0–3	7,573	14,886	7,983	5.3	1.8 (−0.3 to 4.0)	0.09	1.9 (−0.6 to 4.4)	0.13	1.6 (−2.5 to 5.9)	0.44	0.99
	0–4	7,580	14,916	7,988	5.2	1.7 (−0.5 to 3.9)	0.14	1.9 (−0.6 to 4.5)	0.15	1.5 (−2.7 to 5.9)	0.50	0.98
	0–5	7,580	14,938	7,991	5.2	2.2 (−0.1 to 4.5)	0.06	2.6 (−0.1 to 5.4)	0.06	1.3 (−3.1 to 5.9)	0.56	0.88
	0–6	7,581	14,945	7,992	5	2.2 (−0.1 to 4.6)	0.06	2.3 (−0.5 to 5.2)	0.10	0.8 (−3.7 to 5.4)	0.74	0.81
Emergency department visits												
Culture-negative pneumonia	0	23,175	55,434	32,373	6.6	−0.1 (−1.4 to 1.3)	0.93	−0.6 (−1.8 to 0.6)	0.35	1.1 (−0.9 to 3.1)	0.29	0.35
	0–1	23,267	56,319	32,527	6.1	0.9 (−0.5 to 2.3)	0.23	0.2 (−1.1 to 1.5)	0.79	1.2 (−0.9 to 3.4)	0.25	0.59
	0–2	23,353	57,167	32,667	5.8	1.5 (0.0 to 3.1)	0.05	1.0 (−0.3 to 2.3)	0.14	1.6 (−0.7 to 3.9)	0.17	0.82
	0–3	23,360	57,329	32,693	5.3	2.0 (0.4 to 3.6)	0.01	1.5 (0.2 to 2.9)	0.03	1.9 (−0.4 to 4.2)	0.10	0.88
	0–4	23,364	57,433	32,724	5.1	2.9 (1.2 to 4.6)	0.001	1.7 (0.3 to 3.1)	0.02	2.0 (−0.3 to 4.4)	0.10	0.52
	0–5	23,364	57,518	32,741	4.9	3.2 (1.4 to 4.9)	<0.001	2.2 (0.7 to 3.7)	0.004	2.2 (−0.2 to 4.7)	0.08	0.64
	0–6	23,364	57,561	32,754	4.8	3.0 (1.2 to 4.9)	0.001	2.1 (0.6 to 3.7)	0.01	2.0 (−0.5 to 4.5)	0.12	0.69
Influenza	0	4,889	31,115	19,815	6.7	1.5 (−0.9 to 3.9)	0.22	−0.6 (−2.2 to 0.9)	0.42	3.0 (0.5 to 5.6)	0.02	0.04
	0–1	4,931	31,710	19,932	6.4	2.0 (−0.4 to 4.5)	0.10	−0.7 (−2.4 to 1.0)	0.41	3.8 (1.0 to 6.7)	0.01	0.02
	0–2	4,962	32,310	20,036	6.2	3.0 (0.5 to 5.6)	0.02	−0.7 (−2.5 to 1.1)	0.43	5.7 (2.6 to 8.8)*	<0.001	<0.001
	0–3	4,965	32,354	20,046	6	4.1 (1.5 to 6.7)	0.002	0.2 (−1.7 to 2.1)	0.87	5.9 (2.6 to 9.3)*	<0.001	0.01
	0–4	4,966	32,373	20,060	5.9	4.9 (2.3 to 7.6)	<0.001	1.3 (−0.7 to 3.3)	0.22	6.2 (2.7 to 9.8)	<0.001	0.03
	0–5	4,966	32,392	20,072	5.7	5.4 (2.7 to 8.1)	<0.001	2.1 (−0.1 to 4.2)	0.06	6.4 (2.8 to 10.1)	<0.001	0.07
	0–6	4,966	32,404	20,085	5.4	5.4 (2.8 to 8.1)	<0.001	2.7 (0.5 to 4.9)	0.02	5.5 (1.9 to 9.2)	0.002	0.23
Bacterial pneumonia	0	690	3,292	2880	6.2	0.1 (−5.6 to 6.1)	0.98	−0.4 (−4.7 to 4.0)	0.85	7.7 (1.3 to 14.6)	0.02	0.08
	0–1	691	3,338	2896	6	0.1 (−5.9 to 6.5)	0.97	−0.6 (−5.3 to 4.2)	0.79	7.1 (0.2 to 14.4)	0.04	0.15
	0–2	704	3,384	2914	5.2	−0.4 (−6.0 to 5.6)	0.90	−0.3 (−4.8 to 4.5)	0.91	7.2 (0.5 to 14.3)	0.03	0.12
	0–3	704	3,386	2917	5.5	−2.3 (−8.4 to 4.2)	0.48	−1.5 (−6.5 to 3.9)	0.59	6.5 (−1.1 to 14.7)	0.10	0.13
	0–4	704	3,388	2918	5	−2.4 (−8.3 to 4.0)	0.46	−1.1 (−6.3 to 4.3)	0.68	5.2 (−2.2 to 13.2)	0.17	0.22
	0–5	704	3,395	2920	5	−1.7 (−7.9 to 4.9)	0.60	−0.7 (−6.2 to 5.0)	0.80	5.0 (−2.7 to 13.4)	0.21	0.33
	0–6	704	3,396	2920	5	−1.6 (−8.1 to 5.3)	0.64	0.4 (−5.5 to 6.7)	0.90	3.3 (−4.8 to 12.1)	0.44	0.59

*Definition of abbreviations*: CI = confidence interval; IQR = interquartile range.

Note: models adjusted for temperature (4 *df*) and relative humidity using natural splines (4 *df*).

* *P* < 0.025: Test of whether after rate ratio is different from the during rate ratio.

^†^ *P* < 0.05: test of whether after rate ratio is different from during rate ratio.

Although inconsistent with the pattern of relative rates of influenza ED visits, the rate of influenza hospital admissions associated with increased PM_2.5_ concentrations was largest in the before period, with lower rates in the during and after periods. Although not significantly different, the rate of ED visits for bacterial pneumonia was substantially larger in the after period than in the during and before periods. There were no patterns in the relative rate of hospital admissions for bacterial pneumonia (Table 3).

## Discussion

As hypothesized, we found that increased relative rates of hospital admissions and ED visits for culture-negative pneumonia (1–2%) were significantly associated with increased PM_2.5_ concentrations in the previous 2–7 days, whereas increased relative rates of influenza ED visits (3–4%), but not hospital admissions, were associated with increased PM_2.5_ concentrations in the previous 5–7 days. Increased relative rates of bacterial pneumonia hospitalizations were also associated with increased PM_2.5_ concentrations in the previous 1–7 days. These increased relative rates were independent of temperature and relative humidity changes, as well as subject characteristics (e.g., age, race, sex, socioeconomic status, previous health events) that were controlled by design in the case–crossover study.

A few studies have previously reported positive associations between short-term ambient air pollution exposures and hospitalizations for pneumonia (8), outpatient clinic visits for pneumonia (18), viral respiratory infections (5, 6), and general lower respiratory tract infections in subjects with asthma (19), with some associations reported in children (5, 6, 20) and others in adults (5, 8, 19). The Global Burden of Disease estimated a 50–100% increased risk of lower respiratory tract infection associated with 50- to 150-μg/m^3^ increases in PM_2.5_ concentration (9). A recent study of adults in the Wasatch Valley of Utah reported a 7% (95% CI, 4–11%) increased odds of lower respiratory tract infection associated with each 10-μg/m^3^ increase in PM_2.5_ in the previous 7 days (6). These results are consistent with our finding of an ER of hospitalization and ED visits for culture-negative pneumonia (1–2%) and ED visits for influenza (1–4%) (Table 2). The increased association of air pollution with ED visits for influenza in the previous 5–7 days is consistent with the clinical expectation that 95% of patients with influenza become symptomatic within 2 days (21) and may present for care over the next several days as symptoms worsen. A recent large epidemiologic study of respiratory disease and PM_2.5_ in four U.S. cities reported increased ERs (0.6–0.8%) of ED visits for respiratory disease (chronic obstructive pulmonary disease, upper respiratory infection, pneumonia, asthma and/or wheeze, and bronchiolitis) per IQR increase in PM_2.5_ in the previous 2 days. There were similar effect sizes for pneumonia ED visits associated with increased PM_2.5_ concentrations in the previous 1–4 days in each city (10). However, the lack of specificity in the type of infection makes comparisons between studies more difficult. Our inclusion of culture-negative pneumonia (in addition to bacterial pneumonia and influenza) may begin to address this issue. Culture-negative pneumonia is a common clinical diagnosis, due to the limitations in modern culture techniques to accurately establish a microbiologic diagnosis for pneumonia (14). Due to current diagnostic limitations, such as obtaining adequate samples, differentiating infection from colonization, and the difficulty of growing organisms on artificial media, making even a basic differentiation between broad categories of infection (bacterial vs. viral) is difficult (13).

We found the rate of culture-negative pneumonia hospitalizations associated with each 6.2-μg/m^3^ increase in PM_2.5_ concentration in the previous 3 days, and the rate of influenza ED visits associated with each 6.4-μg/m^3^ increase in PM_2.5_ concentration in the previous 3 days, were increased after air quality changes occurred from 2008–2013 (11) compared with the during period. Although concentrations of PM_2.5_ and several other pollutants subsequently decreased, changes in PM composition (e.g., proportionally more SOC) also occurred (12). Similarly, although pollutant concentrations generally decreased in a recent study in Los Angeles, increases in oxidant concentrations were also observed (22). The pattern of PM_2.5_/culture-negative pneumonia associations across time periods suggests that these changes in PM composition and air pollution mixture may indicate that, by some mechanism, the same mass of PM is more toxic in the after period than in earlier periods.

Mechanistically, air pollution’s negative impact on local airways leading to inflammation and disruption of the lung’s innate immune system, including mucociliary clearance, macrophage function, or epithelial barrier disruption, is well studied in cell and rodent models (23–25). The cellular signaling pathways of cytokine-mediated inflammation directed by specific signaling proteins (i.e., Toll-like receptors) appear to be a common response to both air pollution exposure and infectious pathogens, leading to the hypothesis that air pollution can alter the innate immune system’s response to infection (26). A study of diesel exhaust exposure’s effect on human respiratory epithelial cells observed an upregulation of the interferon (*IFN*) gene production via a Toll-like receptor pathway (27), indicating a downstream effect of particulate air pollution on the genetic response to viral infection (28). The increased IFN activity from PM_2.5_ exposure may lead to a priming effect on the immune system, thereby producing a more severe response to viral infections (26). Furthermore, a recent double-blind crossover study of diesel exhaust exposure in humans observed reductions in two antimicrobial peptides (α-defensin 1 and S100A7) in bronchoalveolar lavage fluid, indicating an impaired ability to resolve inflammation from infections (29). The potential for PM_2.5_ to increase the immune/inflammatory response to infection and decrease the ability to clear this inflammation is likely to lead to a severe course of infection requiring an ED visit or hospitalization. Further mechanistic research to elucidate the impact of specific components of PM_2.5_ on the innate immune response to infection is needed.

In our New York State study, SOC is one component observed in higher concentrations in the after period compared with the during period (12). SOCs are a PM_2.5_ component formed from the atmospheric oxidation of biogenic and anthropogenic volatile organic compounds to form reactive particles (30). In the online supplement, we describe the methods for estimating the concentrations of SOC (*see* Equation E1 and Figure E1) and the statistical analysis of the differences in concentrations among the periods (*see* Figures E2 and E3, Tables E3 and E4). Similar to overall PM_2.5_, the broad mechanism of SOC-mediated injury is thought to involve the creation or delivery of reactive oxygen species, leading to oxidative stress and inflammation in the lungs and other organs (31, 32). It is currently unknown to what degree the mechanism of SOC-induced injury overlaps with that of injury from other pollutants. Though speculative, the similar general pattern of relative rates of healthcare encounters for infection observed in our study to the pattern of SOC concentrations (both higher in the after period compared with the during period) may indicate that PM_2.5_ rich in SOC may alter the body’s immune response to infection.

Other possible explanations for the increased relative rates of health care encounters for influenza in the after period in relation to PM_2.5_ compared with earlier periods could be an increase in the virulence of infections, changes in the use or efficacy of the flu vaccine, or an overall change in clinical care. Despite stability in vaccination rates (33), the Influenza Vaccine Effectiveness Network has observed significant variability in the estimated efficacy of the flu vaccine each year (34), thought to be related to genetic changes (antigenic drifts and shifts), which change an individual strain’s virulence (35, 36). The nationwide trend for influenza infection over the course of the during and after periods of our study (2010–2016) was one of increasing rates of hospitalizations and total numbers of medical visits (37). Similarly, across New York State, the proportion of patients hospitalized for influenza infection increased from the before to after period (from 17% to 27%, respectively). Thus, the increasing virulence of influenza infection over time may contribute to the increased relative rate of influenza ED visits associated with PM_2.5_. An explanatory hypothesis is that, even if the impairment of the immune system is lessened in the setting of lower levels of PM_2.5_, the virulence of the organism may lead to a proportionally increased severity in course, increasing the relative rate of clinically significant infection over time. Finally, the trend of increasing numbers of ED visits and decreasing numbers of hospitalizations for culture-negative pneumonia and bacterial pneumonia may reflect an overall change in medical practice, in part due to accountable care organization–driven hospital policies facilitating treatment in the ED and discharge to outpatient care, rather than hospital admission (38).

Although this study could be generalized to most of the eastern half of the United States, where there were decreases in the burning of high sulfur bituminous coal resulting in reductions of SO_2_ concentrations (39), there were also several limitations to consider. First, although multiple national and New York State air quality and energy policies were implemented in the during period (2008–2013), a global recession also occurred in 2008. This economic downturn decreased demand for energy that, in turn, contributed to the reduction in NO_2_ levels in the United States (40). This economic downturn also added to the decreased air pollutant concentrations, due to a slowing of industrial production and reduced energy consumption (41). Thus, any change in the rate of respiratory infection associated with air pollutant concentrations cannot be attributed to individual policies or economic events. Second, all study subjects from each site were assigned the same PM_2.5_ concentrations for a specific day, regardless of how close they lived to a monitoring site, which likely resulted in exposure misclassification. This error is likely a combination of Berkson and classical error, resulting in a bias toward the null and underestimates of effect (42, 43). It is also possible that the observed differences in period-specific rate ratios may be due to differences in the degree of exposure misclassification and underestimation by period (i.e., the ambient PM_2.5_ concentration is a better proxy for individual subject’s PM_2.5_ exposure in the after period than the during period, resulting in a greater underestimation in the during period). Although similar patterns were not observed with all outcomes, future analyses are needed to investigate this further. Third, the diagnosis classification codes were changed mid-study from the 9th version of the ICD (ICD9) to the 10th version (ICD-10) on October 1, 2015 (in the after period). However, all ICD9 and ICD10 codes were reviewed by study physicians to ensure consistency of disease groups included and excluded from the study. Therefore, any outcome misclassification and downward bias should be minimal. To minimize the degree of overlap between case and control periods, we performed a standard case–crossover analysis limited to 7-day lag periods rather than including longer lag periods. In addition, we did not include multiple lag days of temperature and relative humidity in our models, and thus, it is possible that this, in part, could be an explanation for the smaller effect sizes observed at Lag Day 0 compared with other lag days. Finally, SOC values could only be estimated every third or sixth day, depending on the monitoring site, because of the sampling and analysis schedule and time. Thus, a comparable analysis with SOC is not possible with these data.

In summary, increased rates of culture-negative pneumonia healthcare encounters, ED visits for influenza and hospitalizations for bacterial pneumonia were associated with increased concentrations of PM_2.5_ over the previous few days. Changing pollutant mixtures, resulting from air quality policies and decreased energy demand and consumption during the recession, may have changed the toxicity of the PM_2.5_ in this study. The complex relationship between different types of respiratory infections and changing compositions of air pollution mixtures during and after periods of improved air quality requires further study.

## Supplementary Material

Supplements

Author disclosures

## References

[1] Xu J Kochanek KD Murphy SL Arias E Mortality in the United States, 2012 *NCHS Data Brief* 2014 168 1 8 25296181

[2] Ishiguro T Kagiyama N Uozumi R Odashima K Takaku Y Kurashima K *et al* Clinical characteristics of influenza-associated pneumonia of adults: clinical features and factors contributing to severity and mortality *Yale J Biol Med* 2017 90 165 181 28656006 PMC5482296

[3] Jain S Self WH Wunderink RG Fakhran S Balk R Bramley AM *et al* CDC EPIC Study Team Community-acquired pneumonia requiring hospitalization among U.S. adults *N Engl J Med* 2015 373 415 427 26172429 10.1056/NEJMoa1500245 PMC4728150

[4] Pope CA III Dockery DW Health effects of fine particulate air pollution: lines that connect *J Air Waste Manag Assoc* 2006 56 709 742 16805397 10.1080/10473289.2006.10464485

[5] Ciencewicki J Jaspers I Air pollution and respiratory viral infection *Inhal Toxicol* 2007 19 1135 1146 17987465 10.1080/08958370701665434

[6] Horne BD Joy EA Hofmann MG Gesteland PH Cannon JB Lefler JS *et al* Short-term elevation of fine particulate matter air pollution and acute lower respiratory infection *Am J Respir Crit Care Med* 2018 198 759 766 29652174 10.1164/rccm.201709-1883OC

[7] Neupane B Jerrett M Burnett RT Marrie T Arain A Loeb M Long-term exposure to ambient air pollution and risk of hospitalization with community-acquired pneumonia in older adults *Am J Respir Crit Care Med* 2010 181 47 53 19797763 10.1164/rccm.200901-0160OC

[8] Pirozzi CS Jones BE VanDerslice JA Zhang Y Paine R III Dean NC Short-term air pollution and incident pneumonia: a case-crossover study *Ann Am Thorac Soc* 2018 15 449 459 29283681 10.1513/AnnalsATS.201706-495OC

[9] Cohen AJ Brauer M Burnett R Anderson HR Frostad J Estep K *et al* Estimates and 25-year trends of the global burden of disease attributable to ambient air pollution: an analysis of data from the Global Burden of Diseases Study 2015 *Lancet* 2017 389 1907 1918 28408086 10.1016/S0140-6736(17)30505-6 PMC5439030

[10] Krall JR Mulholland JA Russell AG Balachandran S Winquist A Tolbert PE *et al* Associations between source-specific fine particulate matter and emergency department visits for respiratory disease in four U.S. cities *Environ Health Perspect* 2017 125 97 103 27315241 10.1289/EHP271 PMC5226704

[11] Squizzato S Masiol M Rich DQ Hopke PK PM_2.5_ and gaseous pollutants in New York State during 2005–2016: spatial variability, temporal trends, and economic influences *Atmos Environ* 2018 183 209 224

[12] Zhang W Lin S Hopke PK Thurston SW van Wijngaarden E Croft D *et al* Triggering of cardiovascular hospital admissions by fine particle concentrations in New York state: before, during, and after implementation of multiple environmental policies and a recession *Environ Pollut* 2018 242 1404 1416 30142556 10.1016/j.envpol.2018.08.030

[13] Purcaro G Rees CA Wieland-Alter WF Schneider MJ Wang X Stefanuto PH *et al* Volatile fingerprinting of human respiratory viruses from cell culture *J Breath Res* 2018 12 026015 29199638 10.1088/1752-7163/aa9eef PMC5912890

[14] Centers for Disease Control and PreventionNational action plan for combating antibiotic-resistant bacteria. Washington, DC: White House; 20151–63

[15] Maclure M The case-crossover design: a method for studying transient effects on the risk of acute events *Am J Epidemiol* 1991 133 144 153 1985444 10.1093/oxfordjournals.aje.a115853

[16] Levy D Lumley T Sheppard L Kaufman J Checkoway H Referent selection in case–crossover analyses of acute health effects of air pollution *Epidemiology* 2001 12 186 192 11246579 10.1097/00001648-200103000-00010

[17] Aho K Derryberry D Peterson T Model selection for ecologists: the worldviews of AIC and BIC *Ecology* 2014 95 631 636 24804445 10.1890/13-1452.1

[18] Li R Jiang N Liu Q Huang J Guo X Liu F *et al* Impact of air pollutants on outpatient visits for acute respiratory outcomes *Int J Environ Res Public Health* 2017 14 pii: E47 10.3390/ijerph14010047 PMC5295298 28067786

[19] Sinclair AH Edgerton ES Wyzga R Tolsma D A two-time-period comparison of the effects of ambient air pollution on outpatient visits for acute respiratory illnesses *J Air Waste Manag Assoc* 2010 60 163 175 20222529 10.3155/1047-3289.60.2.163

[20] Mehta S Shin H Burnett R North T Cohen AJ Ambient particulate air pollution and acute lower respiratory infections: a systematic review and implications for estimating the global burden of disease *Air Qual Atmos Health* 2013 6 69 83 23450182 10.1007/s11869-011-0146-3 PMC3578732

[21] Lessler J Reich NG Brookmeyer R Perl TM Nelson KE Cummings DA Incubation periods of acute respiratory viral infections: a systematic review *Lancet Infect Dis* 2009 9 291 300 19393959 10.1016/S1473-3099(09)70069-6 PMC4327893

[22] Praske E Otkjær RV Crounse JD Hethcox JC Stoltz BM Kjaergaard HG *et al* Atmospheric autoxidation is increasingly important in urban and suburban North America *Proc Natl Acad Sci USA* 2018 115 64 69 29255042 10.1073/pnas.1715540115 PMC5776813

[23] Harrod KS Jaramillo RJ Rosenberger CL Wang SZ Berger JA McDonald JD *et al* Increased susceptibility to RSV infection by exposure to inhaled diesel engine emissions *Am J Respir Cell Mol Biol* 2003 28 451 463 12654634 10.1165/rcmb.2002-0100OC

[24] Jaspers I Ciencewicki JM Zhang W Brighton LE Carson JL Beck MA *et al* Diesel exhaust enhances influenza virus infections in respiratory epithelial cells *Toxicol Sci* 2005 85 990 1002 15772371 10.1093/toxsci/kfi141

[25] Kaan PM Hegele RG Interaction between respiratory syncytial virus and particulate matter in guinea pig alveolar macrophages *Am J Respir Cell Mol Biol* 2003 28 697 704 12760967 10.1165/rcmb.2002-0115OC

[26] Bauer RN Diaz-Sanchez D Jaspers I Effects of air pollutants on innate immunity: the role of Toll-like receptors and nucleotide-binding oligomerization domain-like receptors *J Allergy Clin Immunol* 2012 129 14 24[Quiz, pp. 25–26.] 22196521 10.1016/j.jaci.2011.11.004 PMC4341993

[27] Ciencewicki J Brighton L Wu W-D Madden M Jaspers I Diesel exhaust enhances virus- and poly(I:C)-induced Toll-like receptor 3 expression and signaling in respiratory epithelial cells *Am J Physiol Lung Cell Mol Physiol* 2006 290 L1154 L1163 16399790 10.1152/ajplung.00318.2005

[28] Kawai T Akira S Innate immune recognition of viral infection *Nat Immunol* 2006 7 131 137 16424890 10.1038/ni1303

[29] Piyadasa H Hemshekhar M Carlsten C Mookherjee N Inhaled diesel exhaust decreases the antimicrobial peptides α-defensin and S100A7 in human bronchial secretions *Am J Respir Crit Care Med* 2018 197 1358 1361 29244524 10.1164/rccm.201708-1714LE

[30] Hopke PK Reactive ambient particles Nadadur S Hollingsworth J Air pollution and health effects London Springer 2015

[31] Xia T Kovochich M Nel A The role of reactive oxygen species and oxidative stress in mediating particulate matter injury *Clin Occup Environ Med* 2006 5 817 836 17110294 10.1016/j.coem.2006.07.005

[32] Tong H Lakey PSJ Arangio AM Socorro J Shen F Lucas K *et al* Reactive oxygen species formed by secondary organic aerosols in water and surrogate lung fluid *Environ Sci Technol* 2018 52 11642 11651 30234977 10.1021/acs.est.8b03695

[33] Williams WW Lu PJ O’Halloran A Kim DK Grohskopf LA Pilishvili T *et al* Centers for Disease Control and Prevention (CDC) Surveillance of vaccination coverage among adult populations—United States, 2014 *MMWR Surveill Summ* 2016 65 1 36 10.15585/mmwr.ss6501a1 26844596

[34] Control CfDPrevention: seasonal influenza vaccine effectiveness, 2005–2015. Atlanta: Centers for Disease Control and Prevention 2015

[35] Bouvier NM Palese P The biology of influenza viruses *Vaccine* 2008 26 D49 D53 19230160 10.1016/j.vaccine.2008.07.039 PMC3074182

[36] Schrauwen EJA de Graaf M Herfst S Rimmelzwaan GF Osterhaus ADME Fouchier RAM Determinants of virulence of influenza A virus *Eur J Clin Microbiol Infect Dis* 2014 33 479 490 24078062 10.1007/s10096-013-1984-8 PMC3969785

[37] Rolfes MA Foppa IM Garg S Flannery B Brammer L Singleton JA *et al*Estimated influenza illnesses, medical visits, hospitalizations, and deaths averted by vaccination in the United States. 2016Dec 9 [accessed 2018 Jul 12]. Available from: https://www.cdc.gov/flu/about/disease/2015-16.htm

[38] McWilliams JM Hatfield LA Chernew ME Landon BE Schwartz AL Early performance of accountable care organizations in Medicare *N Engl J Med* 2016 374 2357 2366 27075832 10.1056/NEJMsa1600142 PMC4963149

[39] Chan EAW Gantt B McDow S The reduction of summer sulfate and switch from summertime to wintertime PM_2.5_ concentration maxima in the United States *Atmos Environ (1994)* 2017 175 25 32 30220859 10.1016/j.atmosenv.2017.11.055 PMC6134864

[40] Russell AR Valin LC Cohen RC Trends in OMI NO2 observations over the United States: effects of emission control technology and the economic recession *Atmos Chem Phys* 2012 12 12197 12209

[41] Tong D Pan L Chen W Lamsal L Lee P Tang Y *et al* Impact of the 2008 Global Recession on air quality over the United States: implications for surface ozone levels from changes in NOx emissions *Geophys Res Lett* 2016 43 9280 9288

[42] Zeger SL Thomas D Dominici F Samet JM Schwartz J Dockery D *et al* Exposure measurement error in time-series studies of air pollution: concepts and consequences *Environ Health Perspect* 2000 108 419 426 10811568 10.1289/ehp.00108419 PMC1638034

[43] Bateson TF Coull BA Hubbell B Ito K Jerrett M Lumley T *et al* Panel discussion review: session three—issues involved in interpretation of epidemiologic analyses—statistical modeling *J Expo Sci Environ Epidemiol* 2007 17 S90 S96 10.1038/sj.jes.7500631 18079770

