# Supplementary material for: The Association between Respiratory Infection and Air Pollution in the Setting of Air Quality Policy and Economic Change
Source: Ann Am Thorac Soc. 2019 Mar;16(3):321–30. doi: 10.1513/AnnalsATS.201810-691OC (PMC6394122; doi:10.1513/AnnalsATS.201810-691OC)
Supplement: Supplements [file AnnalsATS.201810-691OC.html]

The Association between Respiratory Infection and Air Pollution in the Setting of Air Quality Policy and Economic Change | Annals of the American Thoracic Society

- croft\_data\_supplement.pdf (775 KB)
- disclosures.pdf (184 KB)
