# Supplementary material for: The Association between Respiratory Infection and Air Pollution in the Setting of Air Quality Policy and Economic Change
Source: Ann Am Thorac Soc. 2019 Mar;16(3):321–30. doi: 10.1513/AnnalsATS.201810-691OC (PMC6394122; doi:10.1513/AnnalsATS.201810-691OC)
Supplement: Supplements [file AnnalsATS.201810-691OC_croft_data_supplement.pdf]

Supplementary Information for

The Association between Respiratory Infection and Air Pollution in the Setting of Air Quality Policy and Economic Change

Daniel P. Croft MD, MPH[1], Wangjian Zhang PhD[2], Shao Lin MD, PhD[2], Sally W. Thurston, PhD[3], Philip K Hopke, PhD[4,5], Mauro Masiol, PhD[5], Stefania Squizzato, PhD[5], Edwin van Wijngaarden, PhD[4,6], Mark J Utell, MD[1,6], David Q Rich, ScD[1,4,6]

1. *Department of Medicine, University of Rochester Medical Center, Rochester, New York.*
2. *Department of Environmental Health Sciences. University at Albany, the State University of New York, Albany, New York*
3. *Department of Biostatistics and Computational Biology, University of Rochester Medical Center, Rochester, New York*
4. *Department of Public Health Sciences, University of Rochester Medical Center, Rochester, New York*
5. *Institute for a Sustainable Environment, and Center for Air Resources Engineering and Science, Clarkson University, Potsdam, New York*
6. *Department of Environmental Medicine, University of Rochester Medical Center, Rochester, New York*

Table E1. Proportions of hospitalizations and ED visits for respiratory infections by season and period

	Hospitalizations				ED visits			
	**Season**	**Before**	**During**	**After**	**Season**	**Before**	**During**	**After**
**Culture negative pneumonia**	Spring	27.0%	26.0%	27.1%	Spring	26.3%	24.5%	25.4%
	Summer	21.2%	21.7%	22.0%	Summer	19.2%	20.1%	20.6%
	Fall	21.8%	23.1%	22.0%	Fall	23.1%	25.3%	24.2%
	Winter	30.0%	29.1%	28.8%	Winter	31.4%	30.1%	29.8%
**Bacterial pneumonia**	Spring	26.2%	25.4%	26.2%	Spring	26.0%	25.1%	24.3%
	Summer	23.2%	22.5%	22.4%	Summer	20.0%	19.4%	18.8%
	Fall	23.1%	23.6%	23.9%	Fall	25.7%	25.5%	24.3%
	Winter	27.5%	28.6%	27.6%	Winter	28.4%	30.0%	32.6%
**Total Influenza**	Spring	28.3%	18.9%	39.1%	Spring	30.1%	20.3%	39.0%
	Summer	1.2%	8.4%	1.4%	Summer	4.0%	10.7%	1.4%
	Fall	2.4%	11.9%	2.9%	Fall	10.5%	20.0%	4.5%
	Winter	68.0%	60.8%	56.7%	Winter	55.4%	49.0%	55.1%

Separating Organic Carbon into Primary (POC) and Secondary (SOC) Organic Carbon

The premise of the separation of POC and SOC from the OC measurements is that elemental carbon (EC) is only a primary emission. Thus, the lowest ratio values of OC/EC represent the characteristics of primary emissions (Turpin and Huntzicker, 1995). Thus, the SOC can be estimated by

$$SOC = OC - EC * \left[\frac{OC}{EC}\right]_{\mathrm{Pr}i} \qquad \text{(E1)}$$

Where the OC and EC are the measured concentrations and $(OC/EC)_{Pri}$ is the relationship of OC to EC in primary emissions. This ratio is determined by examining the relationship between these variables for the lowest 10th percentile values of the OC/EC ratio. Figure E1 shows the OC –EC plot for the Queens NY site. The resulting slope is then used to estimate the SOC for each sample at each site. POC is then calculated by subtracting the estimated SOC from the measured OC concentration.

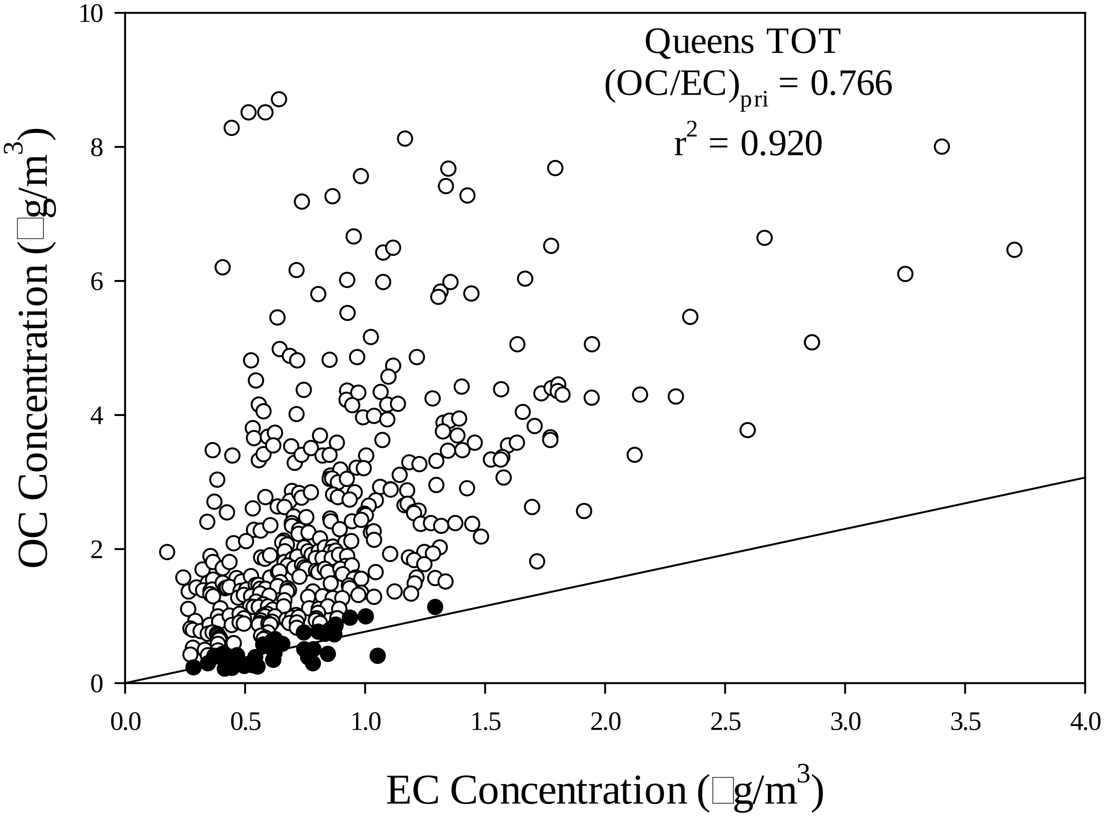

Figure E1. Plot of OC against EC for the data from the Queens, NY NYS DEC site. Filled points represent the lowest 10th percentile values of the OC/EC ratio.

The distributions of SOC and POC for the sites across the study period are shown in Figures E2 and E3, respectively.

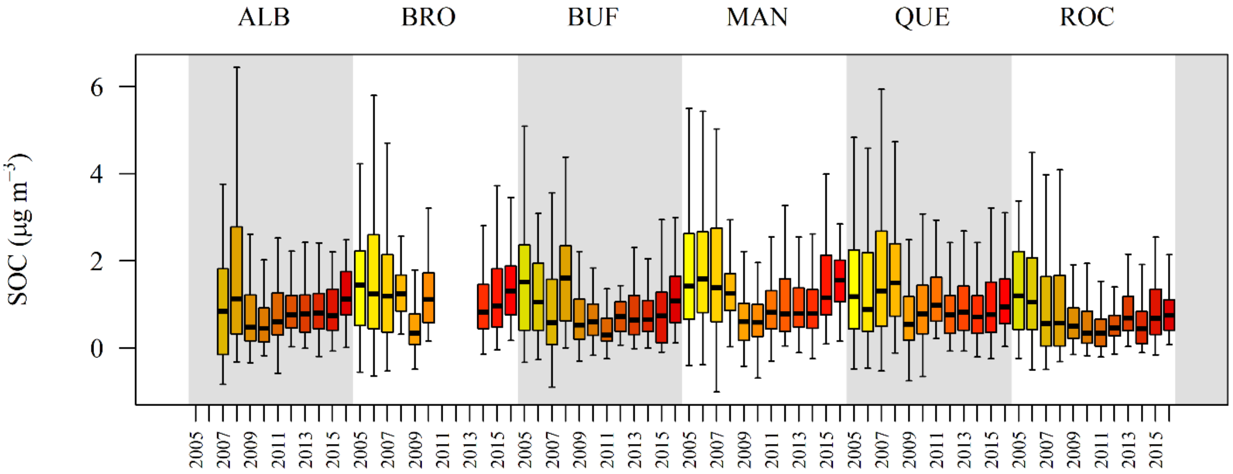

Figure E2. Box and whisker plots of the estimated SOC concentrations at each urban site in NYS by year from 2005 to 2016.

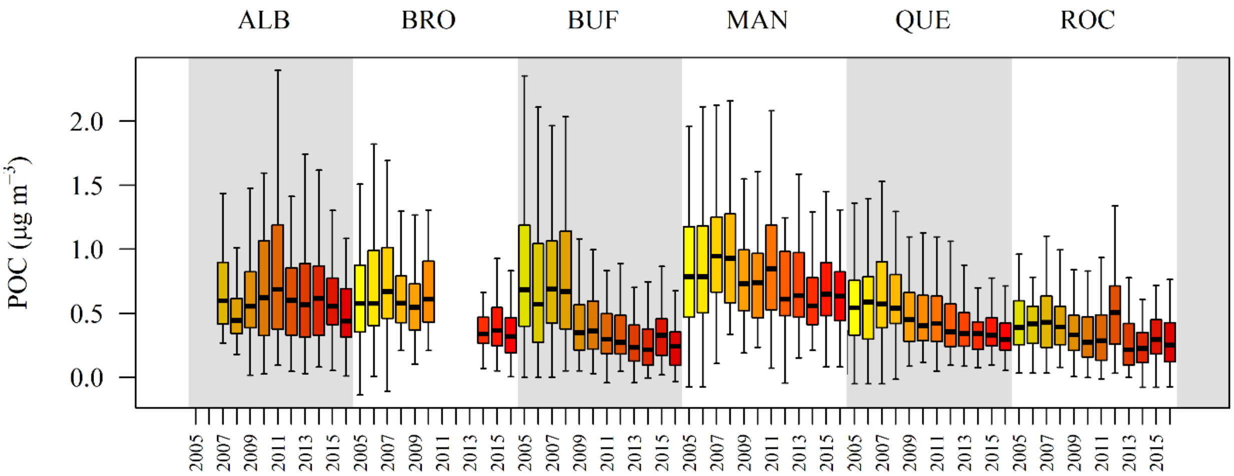

Figure E3. Box and whisker plots of the estimated POC concentrations at each urban site in NYS by year from 2005 to 2016.

To determine the differences among the three periods, a Kruskal-Wallis ANOVA on Ranks analysis was performed for each of these two variables and pairwise analyses using Dunn's test. The results are provided in Tables E1 and E2 for SOC and POC, respectively.

Table E2. Probabilities of differences in median SOC values among periods and pairs of periods. Significant values are bolded

Site	Kruskal-Wallis p-value	Before-During[a] delta (p-value)	During-After delta (p-value)	Before-After delta (p-value)
Albany	**0.005**	-23.3 (1.00)	**-61.7 (0.006)**	-85.0 (<0.162)
Bronx	**<0.001**	**100.7 (<0.001)**	**-66.1 (0.008)**	34.5 (0.264)
Buffalo	**<0.001**	**70.1 (<0.001)**	-28.3 (0.345)	41.8 (0.153)
Manhattan	**<0.001**	**183.1 (<0.001)**	**-127.3 (<0.001)**	55.9 (0.070)
Queens	**<0.001**	**107.7 (<0.001)**	19.0 (1.000)	**126.7 (<0.001)**
Rochester	**<0.001**	**158.0 (<0.001)**	**-86.2 (0.001)**	**71.8 (0.037)**

For Albany, there are significant differences among the three periods, but the only significant pairwise difference was between the During and After periods where SOC increases between these two periods. However, comparisons with the Before period may be biased since this speciation network site did not start until 2007 so there are far fewer samples for the Before period (41) compared to During (625) and After (237). It can be seen that at 5 of the 6 sites, the SOC decreased from Before to During and increased from During to After although not all of the differences were statistically significant.

Table E3. Probabilities of differences in median POC values among periods and pairs of periods. Significant values are bolded

Site	Kruskal-Wallis p-value	Before-During delta (p-value)	During-After delta (p-value)	Before-After delta (p-value)
Albany	0.690	--- [a]	**226.4 (<0.001)**	**197.1 (<0.001)**
Bronx	**<0.001**	29.3 (0.567)	**197.1 (<0.001)**	**226.4 (<0.001)**
Buffalo	**<0.001**	**145.4 (<0.001)**	**69.9 (<0.001)**	**215.3 (<0.001)**
Manhattan	**<0.001**	25.3 (0.815)	**115.9 (<0.001)**	**141.2 (<0.001)**
Queens	**<0.001**	**134.0 (<0.001)**	**159.9 (<0.001)**	**293.9 (<0.001)**
Rochester	**<0.001**	**67.0 (0.023)**	**82.9 (0.001)**	**149.9 (<0.001)**

a. pairwise comparisons only made when K-W shows significant differences

With the exception of Albany, all of the sites had statistically significant differences among the three periods. Again, Albany Before data has very few samples that may have precluded seeing significant differences with the other 2 periods. The pattern for POC is quite different in that the POC decreased from the beginning to the end of the study period.

Reference

Turpin, B.J., Huntzicker, J.J., 1995. Identification of secondary organic aerosol episodes and quantitation of primary and secondary organic aerosol concentrations during SCAQS. Atmos. Environ. 29, 3527e3544.
